# College Matters

**Published:** 1868-08

**Authors:** 


### COLLEGE MATTERS.

At the close of the last session of the Ohio College of Dental Surgery, the chair of Histology and Physiology was made vacant by the resignation of Prof. C. W. Spalding; and subsequently the chair of Practical Dentistry was made vacant by the resignation of Prof. W. T. Arrington.

At a recent meeting of the Board of Trustees of the College, these vacancies were filled ; the former by the election of Prof. R. King Browne, of New York ; and the latter by the election of Dr. J. A. Watling, of Ypsilanti, Michigan.

The mere mention of the name of Prof. Browne in connection with this position, is a sufficient guarantee of the fitness of the appointment. Through the West and North-west, we can say nothing to add to the strength of Dr. Watling's name, for there, none are more generally, or more favorably known.

The readers of the REGISTER will recognize Dr. Watling as the man who some years ago assumed a very decided advance position in the Michigan State Dental Association, on the subject of Dental education, and the Association came up to his standpoint.

In these appointments we anticipate much for the Ohio Dental College. T.

